# External Application of Chloral Hydrate

**Published:** 1871-11

**Authors:** Chas. S. Strother

**Affiliations:** Barnesville, Georgia


### EXTERNAL APPLICATION OF CHLORAL HYDRATE.

BY CHAS. S. STROTHER, M.D., BARNESVILLE, GEORGIA.

I desire to call the attention of the profession to the external application of hydrate of chloral as a powerful and speedy counter-irritant and, to a considerable extent, a local anodyne. The idea first suggested itself to my partner, Dr. McDowell, during the summer of 1870, when, on one occasion, he accidentally applied a small portion of chloral to his own face, instantly producing a burning sensation.

A few nights subsequent to this occurrence he was called to visit a case of bilious colic (so called.) He immediately prepared and applied a saturated solution of chloral to the stomach and bowels of the sufferer, with the most happy effect.

We have since used it with good results in facial neuralgia, pleurodynia, rheumatism, gastralgia; in uterine and ovarian pains; in the acute suffering of advanced phthisis; and last, but not least, we have found it more efficient in relieving persistent nausea and vomiting than any other remedy we have ever used. In one case of the latter trouble, after administering calomel, sol. atropia, tinct. aconite, sub. nit. bismuth, creosote, oxalate of cereum, lime water, tinct. columbo, carbolic acid, morph. sulph. used hypodermically, blisters and sinapisms in vain, imagine our joy to see our patient speedily relieved by the external application of hydrate of chloral.

We have used this remedy, applied as above, in *numerous* cases of nausea and vomiting, and have never yet been disappointed. Of course we do not claim for this medicine that it will remove the cause of the vomiting, but it *will* check the vomiting and soothe the patient, and give other appropriate remedies an opportunity to accomplish what they otherwise would have been unable to have done.

The mode of applying the chloral which we have employed is as follows: Place ℈j. in a plate or a saucer; add water enough to make a saturated solution; then apply with the tips of the fingers, with slight friction, over a space as large as the palm or *pro. re nata.* Should the burning be too intense, the parts may be sponged off in a few minutes with a cloth wrung out of warm water; and then apply glycerine, with a little spirits camphor, or the glycerine alone, olive oil, or sweet cream.

The skin assumes a red and rather a parched appearance, and there will be some irritation consequent upon this treatment, but never enough to deter us from repeating the application, should it become necessary.

There will, in many or *most* instances, be enough of the chloral absorbed to produce a considerable anodyne effect; therefore, it would be well to avoid giving more than half the usual dose per orem, if given at all, immediately after using it externally.

